# Supplementary material for: The causal relationship between the human gut microbiota and pyogenic arthritis: a Mendelian randomization study
Source: Front Cell Infect Microbiol. 2024 Nov 26;14:1452480. doi: 10.3389/fcimb.2024.1452480 (PMC11629706; doi:10.3389/fcimb.2024.1452480)
Supplement: Supplementary file 1 [file DataSheet1.zip › mendelian test/ebi-a-GCST90027550.csv_forest.pdf]

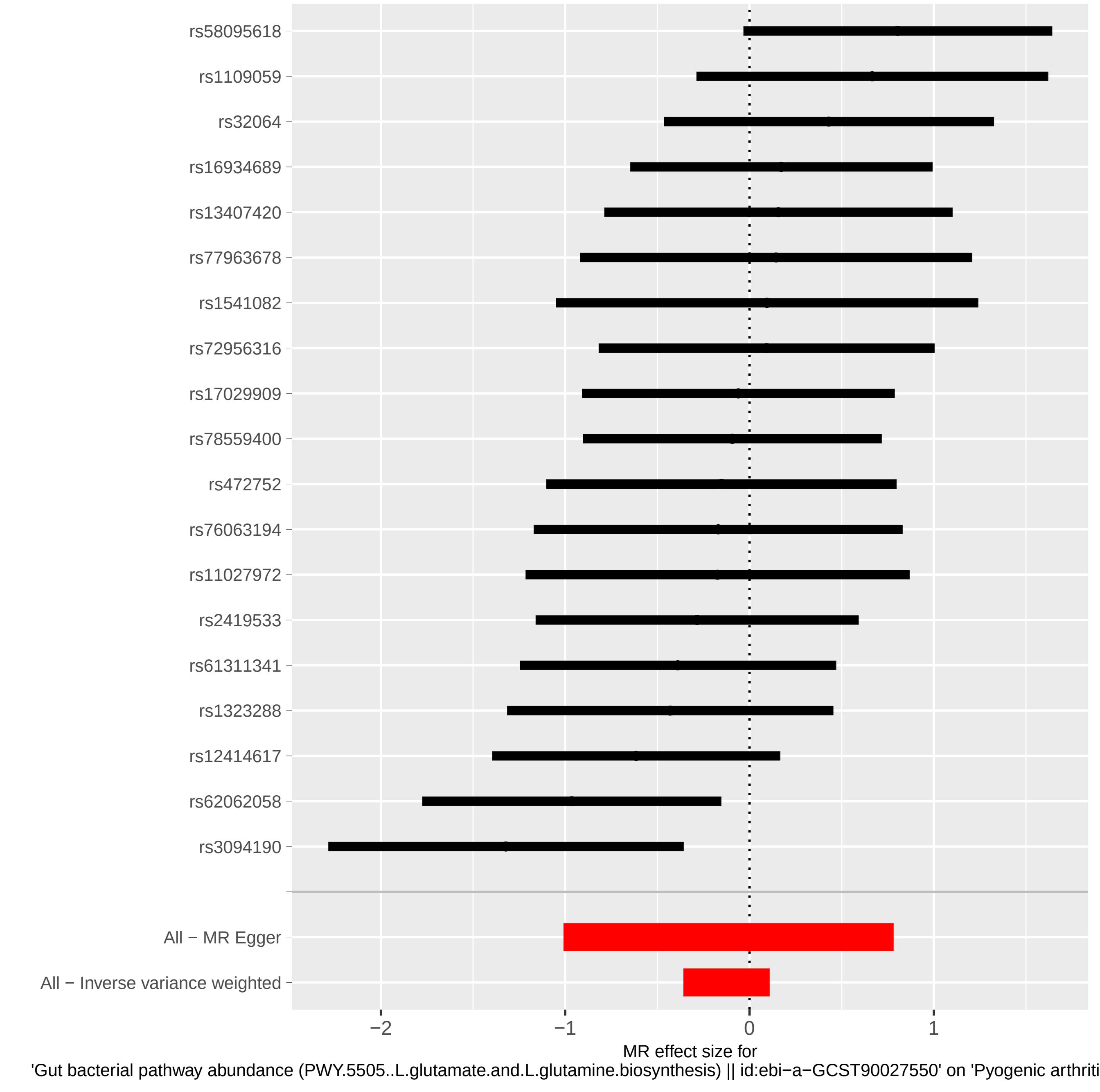

rs58095618
rs1109059
rs32064
rs16934689
rs13407420
rs77963678
rs1541082
rs72956316
rs17029909
rs78559400
rs472752
rs76063194
rs11027972
rs2419533
rs61311341
rs1323288
rs12414617
rs62062058
rs3094190
All − MR Egger
All − Inverse variance weighted
−2
−1
0
1
MR effect size for
'Gut bacterial pathway abundance (PWY.5505..L.glutamate.and.L.glutamine.biosynthesis) || id:ebi−a−GCST90027550' on 'Pyogenic arthriti
